# Fluorescence Studies of the Interplay between Metal-Enhanced Fluorescence and Graphene-Induced Quenching

**DOI:** 10.3390/ma11101916

**Published:** 2018-10-09

**Authors:** Kamil Wiwatowski, Paweł Podlas, Magdalena Twardowska, Sebastian Maćkowski

**Affiliations:** Institute of Physics, Faculty of Physics, Astronomy and Informatics, Nicolaus Copernicus University, Grudziądzka 5, 87-100 Torun, Poland; kamilw@doktorant.umk.pl (K.W.); 259046@stud.umk.pl (P.P.); magda@fizyka.umk.pl (M.T.)

**Keywords:** graphene, silver island film, metal-enhanced fluorescence, energy transfer

## Abstract

Fluorescence microscopy and spectroscopy were applied for studying the optical properties of a hybrid nanostructure, in which we combine plasmon-induced metal enhanced fluorescence with energy transfer to epitaxial graphene. Covering the layer of silver islands with a monolayer graphene, while turning on the efficient energy transfer from emitters, only moderately affects the enhancement of fluorescence attributed to the plasmon resonance in metallic nanostructures—as evidenced by the analysis of fluorescence decays. The results show that it is feasible to combine the properties of graphene with metal-enhanced fluorescence. The importance of the layer thickness of the emitters is also pointed out.

## 1. Introduction

At the nanoscale, bringing two or more nanostructures close together may frequently lead to interactions between them, which in turn induce modifications in the properties of such fabricated hybrid structures [1]. By proper design thereof, it is possible to improve the desired properties or even add new functionalities, minimizing at the same time any effects considered parasitic for a particular application. Among the interactions that emerge at the nanoscale, there are two that arguably are the most prominent: Metal-enhanced fluorescence (MEF) [2] and fluorescence resonance energy transfer (FRET) [3].

The MEF effect is a process, in which the fluorescence intensity of an emitter is increased via interactions with plasmonic excitations in metallic nanoparticles placed in its vicinity [2,4,5]. A source of this enhancement is an increase of the radiative emission rate of the emitter, although the emission intensity may also increase as a result of plasmonically improved absorption. The strength of MEF depends on the emitter-nanoparticle distance, as well as their spectral matching, thus optimization of these parameters is important to achieve strong fluorescence enhancement [6,7,8].

Plasmonically active metallic nanostructures can be either based on nanoparticles, synthesized chemically [9], or planar structures, such as Silver Island Film (SIF) [10]. While the control of the morphology is rather difficult in the latter, the enhancements observed for emitters deposited on SIF have been considerable [11], as demonstrated for many different emitters including semiconductor nanocrystals [5], organic dyes [12], and biological photosynthetic complexes [10].

On the other hand, the FRET process is associated with transferring the energy between two nanostructures [3]. This non-radiative process results in quenching of donor fluorescence intensity together with the shortening of the donor fluorescence lifetime. The basic requirement for FRET to occur is spectral overlap between donor emission and absorption of the acceptor [6]. In addition, the efficiency of FRET strongly depends on the distance between donor and acceptor [6]. In this context, graphene, a monoatomic layer of carbon atoms arranged in a hexagonal lattice [13], is a unique energy acceptor. Namely, as it exhibits constant absorption across the whole visible and infrared spectral ranges, which is equal to 2.3% of incident light [14], the spectral overlap requirement is directly achieved. In contrast to the classic case of two molecules, in the case of graphene the distance dependency of the FRET efficiency is equal to d^−4^ [15], as confirmed by Koppens and coworkers. The efficiencies of the energy transfer to graphene are usually very high, amounting often to 90% or more [16]. Is has also been shown that the efficiency of the energy transfer is affected by morphology of graphene, as well as the illumination conditions [17,18].

In this work, we study the interplay between MEF and FRET effects in a hybrid nanostructure composed of SIF covered with graphene, on top of which a layer of emitters was deposited. Fluorescence microscopy and spectroscopy allowed us to conclude that covering the plasmonically active layer with graphene is not inhibiting the MEF effect. At the same time, we find an increase in inhomogeneity of fluorescence intensity for graphene-covered SIF layers with a partial correlation between the intensity and fluorescence decays.

## 2. Materials and Methods

Silver Island Films were deposited on glass coverslips by reducing silver nitrate with D-glucose, using the protocol described previously [19]. The morphology of the SIF layer was previously examined using Scanning Electron Microscopy. The results have shown that SIF is rather inhomogeneous with the size of the islands ranging from a few tens of nanometers up to 100 nm. SIF structure is perfectly suitable for experiments described in this work since it can be easily fabricated over large substrates and the SIF layer itself is not affected by the solvents used for transfer of graphene. 

As graphene material, we used Single-Double layer CVD (Chemical Vapor Deposition). graphene (SDG) grown on a copper foil, these substrates were purchased from Graphene Supermarket (Graphene Laboratories Inc., Calverton, NY, USA). These graphene structures are characterized by having over 70% of the surface of monolayer graphene, with the rest being a bilayer structure. To transfer graphene on either glass or SIF substrate, we used an approach based on Polymer-Assisted-Transfer method described in [20]. As a handling layer for graphene, we used 2% cellulose nitrate from Sigma-Aldrich (Saint Louis, MO, USA). To improve the quality and purity of the graphene layer the polymer-graphene stack was washed using hydrochloric acid and ammonia solution. Finally, acetone was used to dissolve polymer, thus leaving graphene on either glass or SIF substrate. This approach requires no specialized equipment and results in a high quality of graphene layers.

**Figure 1:**
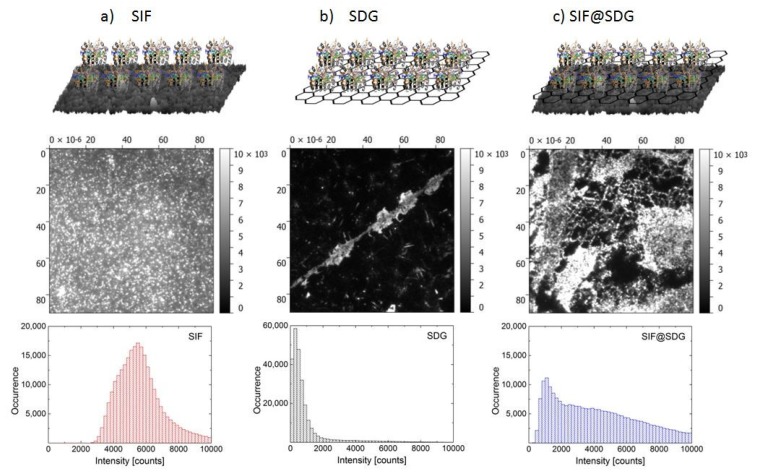
Fluorescence imaging of three studied structures composed of PCP complexes deposited on (**a**) Silver Island Film on glass; (**b**) Single-Double layer graphene on glass; and (**c**) Single-Double layer graphene transferred on Silver Island Film. For each structure we show a typical fluorescence map and corresponding intensity histogram obtained with the excitation at 480 nm. The intensity scale is the same for all fluorescence images.

As a fluorescence probe, Peridinin-Chlorophyll-Protein (PCP) photosynthetic complex was used. It is a simple light-harvesting, water-soluble complex from *Amphidinium carterae*. In its native form PCP is a trimer and its structure was determined using X-ray crystallography [21]. In this work, we used reconstituted PCP complexes, which feature a monomer structure composed of two similar subunits, each containing 4 peridinins, 1 lipid molecule, and 1 chlorophyll *a* molecule. All pigments are embedded in a protein matrix, which shields the pigments from the environment. PCP has broad absorption, spanning from 400 to 650 nm, which strongly overlaps with the plasmon resonance of SIF [10]. The emission of PCP appears at 673 nm. It has been shown that the optical properties of PCP can be strongly affected by both the presence of SIF [10] and graphene [17], where, respectively, strong enhancement of fluorescence intensity and dramatic quenching of the emission was observed. For sample preparation, 30 µL of 50 µg/mL solution of PCP in 0.2% polyvinyl alcohol (PVA from Sigma-Aldrich) was spin-coated at 17 rps on SIF substrate (SIF), graphene (SDG), and SIF coated with graphene (SDG@SIF). Schematic images of the structures studied in this work are displayed in Figure 1.

Fluorescence intensity maps were measured using Nikon Eclipse Ti-U (Melville, LA, USA) inverted wide-field fluorescence microscope equipped with LED illuminators and Andor iXon Du-888 EMCCD camera (Belfast, UK). Excitation beam from a LED illuminator, giving the excitation wavelength of 480 nm, was directed by a dichroic mirror T650lpxr (Chroma, Bellows Falls, VT, USA) to a microscope objective (LPlan, 100×, NA = 0.7). In order to avoid issues associated with limited absorption of both graphene and SIF, the experiments were carried out without immersion oil, and the samples were illuminated from the side of the PCP layer. The PCP fluorescence was detected by the same objective and filtered using a combination of FEL650 edge (ThorLabs, Newton, NJ, USA) and ET675/20 bandpass (Chroma) filters. Fluorescence intensity maps with sizes of 90 × 90 µm were measured with typical parameters of electron multiplying gain and an acquisition time of 1000 and 1 s, respectively. In order to assure for sufficient statistics, for each structure 20 fluorescence maps were measured on different locations across the sample. 

Time and spectrally-resolved measurements were performed using a home-built scanning confocal fluorescence microscope. For excitation of PCP emission we used a 485 nm laser with a repetition rate of 20 MHz and power of 1 µW. The excitation beam was focused on the sample using a microscope objective (LPlan Apo VC, 60×, NA = 1.2, Nikon, Tokyo, Japan). The same objective was used to collect the fluorescence signal, which was then directed through a confocal aperture and an edge HQ655LP filter (Chroma) to our detection system. First, for each of the three structures, a fluorescence map was collected, by correlating the movement of a piezoelectric stage with signal readout from SPCM-AQRH-16 avalanche photodiode (PerkinElmer, Waltham, MA, USA). For measuring fluorescence intensity maps we used an additional bandpass filter ET675/20 (Chroma). In the next step a series of fluorescence decay curves were acquired for approximately 100 spots across the map with time-correlated single photon counting module (SPC-150 Becker & Hickl, Berlin, Germany) and the same combination of optical filters. Detection was provided by a fast avalanche photodiode (idQuantique id100-20, idQuantique, Geneva, Switzerland) Finally, fluorescence spectra were collected with Andor iDus DV 420A-BV CCD camera coupled with an Amici prism. In this way, complete information about the optical properties of fabricated hybrid nanostructures can be obtained, and the effects of both metal-enhanced fluorescence and energy transfer can be elucidated. 

## 3. Results and Discussion

In Figure 1, we show typical fluorescence intensity maps measured for PCP deposited on: (a) SIF layer, (b) graphene, and (c) SIF covered with graphene. In addition, we also present schematic drawings of the structures. The emission map measured for PCP deposited directly on a glass substrate (not shown) features highly homogeneous intensity distribution over the whole map. Fluorescence intensity maps of PCP placed on SIF (Figure 1a) feature high emission intensity, with many bright spots. The histogram of fluorescence intensity extracted from this map is also shown. The distribution is rather broad, which may indicate variation of both the sizes and shapes of the silver islands, as well as changes of the distance between PCP complexes and silver islands. All these factors determine the strength of the interaction, and the resulting intensity of fluorescence emission, associated with metal-enhanced fluorescence. This result agrees qualitatively with previous experiments [10]. In contrast, upon depositing a layer of PCP on graphene (Figure 1b) we observed a strong reduction of the emission intensity in comparison with the result obtained for the SIF structure. Indeed, as can be seen on the corresponding histogram, the maximum emission intensities are on average over six-fold less. The decrease of fluorescence emission intensity results from the energy transfer from PCP complexes to graphene. This interpretation is further confirmed by the results of time-resolved fluorescence discussed later. The fluorescence map also reveals the presence of regions characterized with high emission intensity, which contributes to the high-intensity tail visible in the histogram. These intensities are, however, still less than those measured for PCP on the SIF layer. Such a behavior has been observed previously and has been attributed to either cracks in the graphene layer or regions with a bilayer graphene, which exhibits lower efficiency of the energy transfer [17]. Such local inhomogeneities in graphene morphology induce some broadening of the fluorescence intensity histogram, although their contribution is limited. The combination of both effects: Metal-enhanced fluorescence driven by plasmon excitations in SIF and graphene-induced quenching is at full display in Figure 1c, where the fluorescence map for the SDG@SIF structure is shown. The image is very inhomogeneous with many bright regions mixed with areas of lower intensity. The histogram extracted from this fluorescent map confirms that the intensities of PCP emission cover the whole range: From the values observed for PCP on graphene to the values observed for PCP on SIF. 

**Figure 2:**
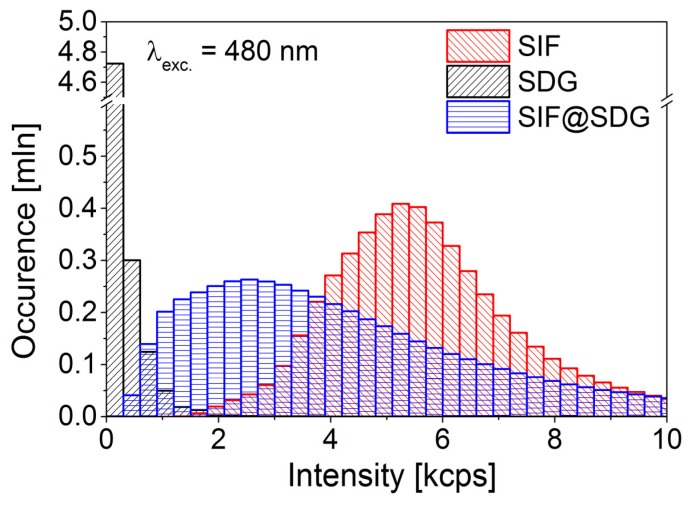
Fluorescence intensity histograms obtained from 10 maps measured for each studied sample: PCP on SIF (red), PCP on SDG (black), and PCP on SDG@SIF (blue). The excitation wavelength was 480 nm.

While the results obtained for a single map (as those shown in Figure 1) bear limited information, which can be obscured, for instance, by selecting some particular region of the sample, adding all the data together allows to gain more conclusive and statistically relevant information. In Figure 2 we show fluorescence intensity histograms for PCP deposited on SIF, graphene, and SDG@SIF with the values gathered from all of the maps acquired in the experiment. The lowest values are obtained for PCP complexes deposited on graphene, with the average value of 140 cps. This indicates fluorescence quenching due to efficient energy transfer from the photosynthetic complexes to graphene. Importantly, the tail towards high-intensity values is less prominent in this case. On the other hand, the average fluorescence intensity obtained for PCP complexes deposited on SIF is equal to almost 6000 cps. Furthermore, the distribution is rather broad, again indicating variation of parameters that govern the strength of the plasmon interaction and the resulting metal-enhanced fluorescence signal. The most interesting behavior is found for the PCP complexes deposited on SDG@SIF substrate, where the interplay between metal-enhanced fluorescence and energy transfer induced quenching is expected. The values of fluorescence intensity measured for this sample are between those measured for the two other samples, PCP on SIF, and PCP on graphene. While the maximum of the distribution can be found around 2500 cps, the average value is around 4000 cps, indicating highly non-symmetrical distribution, with substantial shift towards high-intensity values. Such a large broadening is a combination of inhomogeneities associated with variations of the energy transfer efficiency across non-perfect graphene layer and of the plasmon induced metal-enhanced fluorescence. These results indicate that, despite covering the SIF layer with graphene, the plasmon interaction associated with metallic nanoparticles is strong enough to still influence the optical properties of the emitters [22].

**Figure 3:**
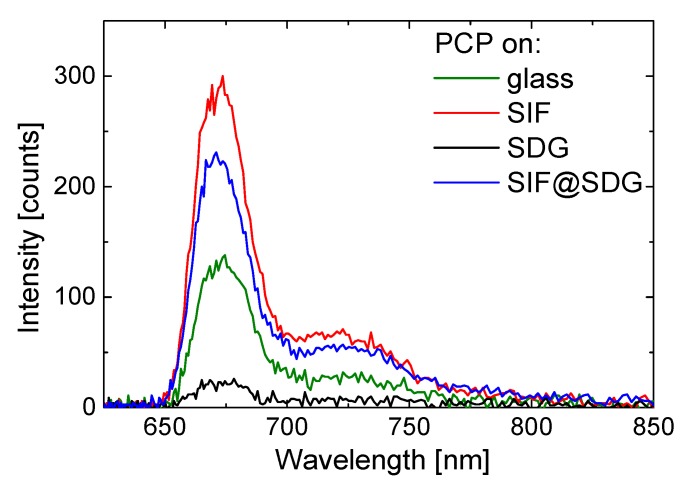
Fluorescence spectra measured for PCP on SIF (red), PCP on SDG (black), and PCP on SDG@SIF (blue). In addition the spectrum measured for PCP on glass is shown in green. The excitation wavelength was 485 nm.

While wide-field fluorescence microscopy gives information about intensities, confocal microscopy provides details about excitation dynamics in hybrid nanostructures, as it gives access to spectrally and time-resolved fluorescent properties. In Figure 3, we compare fluorescence spectra for SIF, SDG, and SDG@SIF samples with the one measured for PCP deposited on a glass substrate. The spectra were measured after collecting a fluorescence map for each sample. In the Supplementary Information Section, we include all of the spectra measured in this experiment. Importantly, wide-field and confocal microscopy results are qualitatively identical regarding fluorescence intensity maps. For each sample, we are able to collect the spectrum that resembles the spectrum of PCP complexes [23]. This indicates that the protein is indeed present and is optically functional.

**Figure 4:**
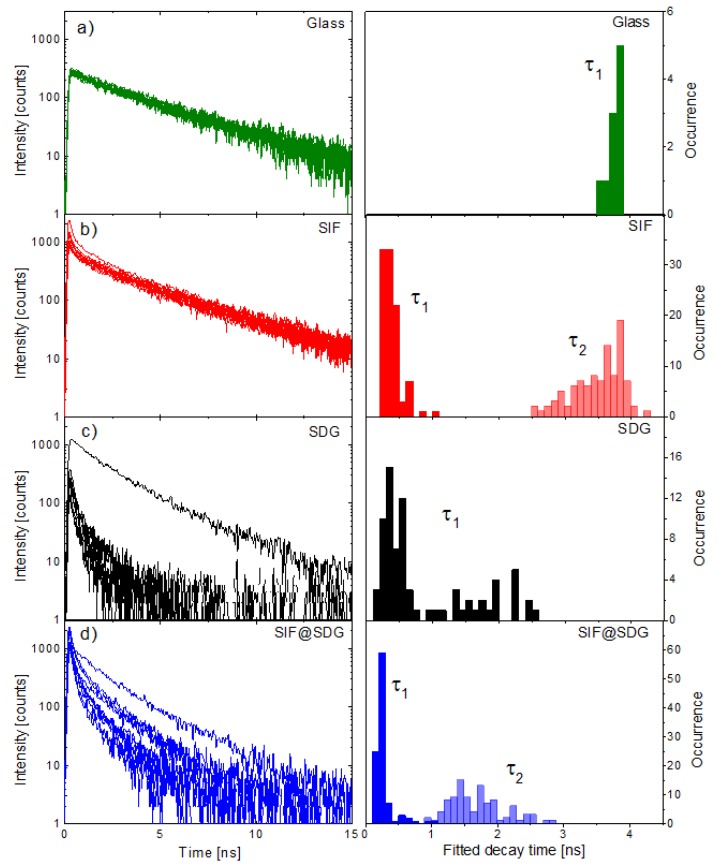
Examples of fluorescence decay curves and histograms of fitted decay times for each fo the investigated samples: (**a**) PCP on glass (green); (**b**) PCP on SIF (red); (**c**) PCP on SDG (black); and (**d**) PCP on SDG@SIF (black). The excitation wavelength was 485 nm.

In Figure 4 we display the results of time-resolved spectroscopy experiments carried out for SIF, SDG, and SDG@SIF structures and compared them with the transients measured for the reference, that is, PCP on a glass substrate. In addition to typical fluorescence decay curves, we also present the results of fitting the transients with appropriate number of decay constants. For PCP complexes deposited on a glass substrate the decay is monoexponential with the average fluorescence decay time equal to 3.8 ns (Figure 4a). Placing the PCP complexes on SIF substrate yields biexponential behavior of the fluorescence transient (Figure 4b). Fitting the decay with such a dependence results in a long-living component (τ_2_) equal, approximately, to the average decay time measured for PCP on glass substrate (Figure 4a,b). It is accompanied with a much shorter decay, which amounts to 0.5 ns (τ_1_) and is consistent with previously reported results [10]. While the emission characterized with the long decay time can be attributed to the PCP complexes that are not interacting with plasmon excitations in the SIF layer, presumably because they are too far apart from the silver islands, the short decay corresponds to the emission of the PCP complexes that couple with the plasmon excitations in the SIF. This coupling leads to an increase of the radiative rate of chlorophyll emission, and is similarly observed for a variety of other emitters. Another process that might lead to the increase of fluorescence emission, but with no effect on the fluorescence decay, is increased by the absorption rate, which can also take place in the studied structure. As the optimal distance between emitters and metallic nanoparticles for the MEF effect is in the range of 10–20 nm [7], it is reasonable to assume that the thickness of the PVA layer containing PCP complexes is relatively large, of the order of 50 or more nm. From the comparison of the decay times, it can be suggested that the increase of the emission intensity of PCP complexes interacting with the plasmon excitations in SIF is about eightfold. Fluorescence quenching, observed for the PCP complexes deposited on graphene, is a result of the energy transfer (Figure 4c), as evidenced by dramatic shortening of the decay time. With rather good accuracy the decays measured for PCP on graphene can be approximated with a single exponential decay. The values of the decay times are very short, mostly less than 1 ns, which translates to the efficiency of the energy transfer of about 80%. Notably, we do not find in this case a long-living component, which—in analogy with the SIF structure—would have been attributable to the PCP complexes that exhibit no interaction with graphene. It is perhaps due to much thinner layer of the PCP complexes on a graphene substrate, which is more hydrophobic and much flatter than the SIF substrate. On the other hand, since the dependence of the efficiency of the energy transfer to graphene is inversely proportional to the fourth power of the distance, even if the thickness of the PCP-containing PVA layer is about 50 nm, most of the PCP complexes will interact with graphene. At the same time, we also find decays like the one shown in Figure 4c, which was measured for the aggregate of the PCP complexes. In such cases no shortening of fluorescence decay was observed. Finally, the sample combines SIF and graphene features intermediate behavior of fluorescence decays, which can be approximated with two decay constants (Figure 4d). The shorter component is the shortest of all of the decays measured, which can be attributed to the PCP complexes that experience both, plasmon enhancement and energy transfer to graphene. On the other hand, the complexes, whose fluorescence exhibits decay times in the range of 1.5 ns can be assigned to the fraction of photosynthetic proteins that experienced limited influence of the plasmon excitations in SIF structures, but participate in energy transfer to graphene. Based on these results, it is possible to speculate that the thickness of the PCP-containing PVA layer is in the case of this structure also intermediate in comparison with SIF and SDG structures. In other words, placing graphene onto the SIF layer smoothens the surface to some degree, making the layer of PCP thinner, which in turn increases the fraction of PCP complexes that interact both with plasmon excitations in SIF and transfer their energy to graphene. On the other hand, the interaction with plasmon excitations in SIF is—despite covering with graphene—still strong enough to influence the optical properties of nearby emitters.

**Figure 5:**
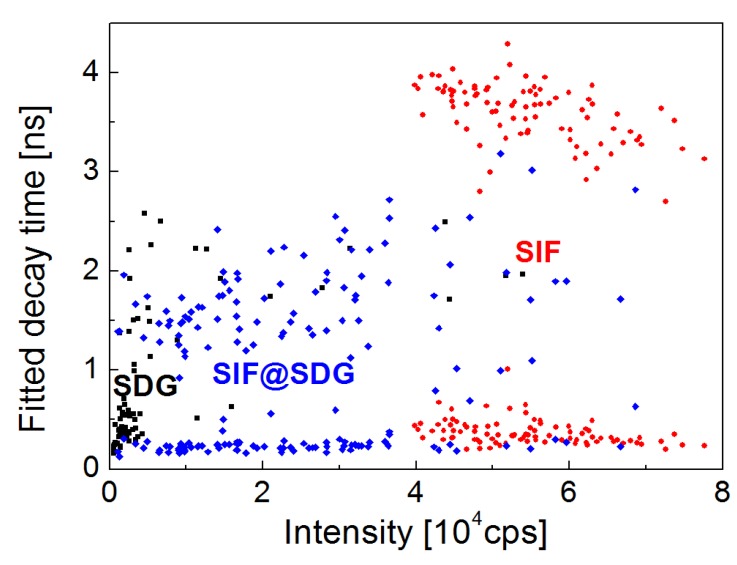
Correlation plot showing fluorescence intensities of PCP emission obtained by integrating all measured transient curves as a function of decay times fitted to these transients. Data for PCP on SIF (red), PCP on SDG (black), and PCP on SDG@SIF (blue) are shown. The excitation wavelength was 485 nm.

The results of fluorescence dynamics studies are summarized in Figure 5, where we show correlation plot relating integrated fluorescence intensities and respective decay times. This comparison shows, in a clear way, that the optical characteristics observed for the structure, where the SIF layer is covered with graphene (blue points) locate themselves between the results extracted for PCP complexes deposited on SIF substrates (red points) and on graphene (black points). The datapoints obtained for these two samples are rather narrowly distributed, both in terms of intensities and decay times. This summary plot shows that bringing together plasmonic structure (SIF) and the energy acceptor (graphene) leads to emergence of spectroscopic signatures of both metal-enhanced fluorescence facilitated by SIF, and efficient energy transfer facilitated by the SDG layer.

## 4. Conclusions

We assembled a hybrid nanostructure, which combined the benefits of plasmonic properties characteristic for metallic nanostructures, with unique behavior of graphene as an energy acceptor. The interplay between metal-enhanced fluorescence and fluorescence resonant energy transfer is demonstrated by fluorescence imaging and time-resolved spectroscopy. While the decay time of emitters deposited on SDG@SIF is shorter than for the SIF structure, the intensity decrease is moderate, indicating that the plasmon interactions is still affecting the optical properties of the emitters. This result opens way of exploiting the unique properties of graphene, and also in the context of plasmonically active metallic nanostructures.

## Supplementary Materials

The following are available online at http://www.mdpi.com/1996-1944/11/10/1916/s1, Figure S1: Fluorescence spectra measured for PCP complexes deposited on all four substrates.
