# Supplementary material for: Fluorescence Studies of the Interplay between Metal-Enhanced Fluorescence and Graphene-Induced Quenching
Source: Materials (Basel). 2018 Oct 9;11(10):1916. doi: 10.3390/ma11101916 (PMC6212895; doi:10.3390/ma11101916)
Supplement: Supplementary file 1 [file materials-11-01916-s001.pdf]

Article

Fluorescence Studies of the Interplay between Metal-Enhanced Fluorescence and Graphene-Induced Quenching

Kamil Wiwatowski, Pawel Podlas, Magdalena Twardowska, Sebastian Maćkowski *

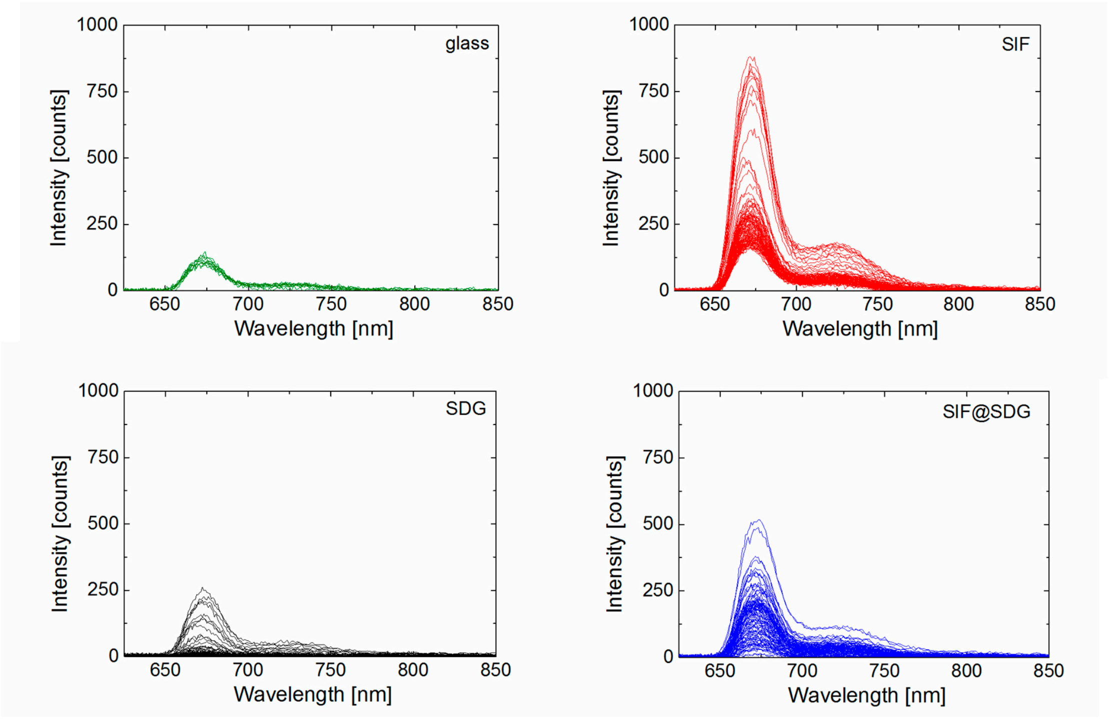

Figure S1. Fluorescence spectra measured for PCP complexes deposited on all four substrates.
